# Drug‐induced anaphylaxis in the emergency room

**DOI:** 10.1002/ams2.282

**Published:** 2017-05-15

**Authors:** Tomonori Takazawa, Kiyohiro Oshima, Shigeru Saito

**Affiliations:** ^1^ Intensive Care Unit Gunma University Hospital Maebashi Gunma Japan; ^2^ Department of Emergency Medicine Gunma University Graduate School of Medicine Maebashi Gunma Japan; ^3^ Department of Anesthesiology Gunma University Graduate School of Medicine Maebashi Gunma Japan

**Keywords:** Cardiopulmonary arrest, emergency room, shock

## Abstract

Anaphylaxis is a life‐threatening, systemic allergic reaction that presents unique challenges for emergency care practitioners. Anaphylaxis occurs more frequently than previously believed. Therefore, proper knowledge regarding the epidemiology, mechanisms, symptoms, diagnosis, and treatment of anaphylaxis is essential. In particular, the initial treatment strategy, followed by correct diagnosis, in the emergency room is critical for preventing fatal anaphylaxis, although making a diagnosis is not easy because of the broad and often atypical presentation of anaphylaxis. To this end, the clinical criteria proposed by the National Institute of Allergy and Infectious Diseases and the Food Allergy and Anaphylaxis Network are useful, which, together with a differential diagnosis, could enable a more accurate diagnosis. Additional *in vitro* tests, such as plasma histamine and tryptase measurements, are also helpful. It should be emphasized that adrenaline is the only drug recommended as first‐line therapy in all published national anaphylaxis guidelines. Most international anaphylaxis guidelines recommend injecting adrenaline by the intramuscular route in the mid‐anterolateral thigh, whereas i.v. adrenaline is an option for patients with severe hypotension or cardiac arrest unresponsive to intramuscular adrenaline and fluid resuscitation. In addition to the route of administration, choosing the appropriate dose of adrenaline is essential, because serious adverse effects can potentially occur after an overdose of adrenaline. Furthermore, to avoid future recurrence of anaphylaxis, providing adrenaline auto‐injectors and making an etiological diagnosis, including confirmation of the offending trigger, are recommended for patients at risk of anaphylaxis before their discharge from the emergency room.

## Introduction

Anaphylaxis is defined as a serious allergic reaction with a rapid onset, which can be fatal.^1^ In an emergency room (ER) setting, it is usually difficult for physicians to correctly diagnose anaphylaxis because of its broad and often atypical presentation. Moreover, the onset of symptoms and suspected allergens are unknown in most cases. These factors often lead to failure in the recognition of anaphylaxis. In fact, studies have shown that a large percentage of patients who present to the ER with anaphylaxis are misdiagnosed.^2^, ^3^, ^4^ In this review, we seek to highlight the current knowledge regarding the epidemiology, clinical presentation, diagnosis, and treatment of anaphylaxis in an ER setting.

## Epidemiology

**Figure 1:**
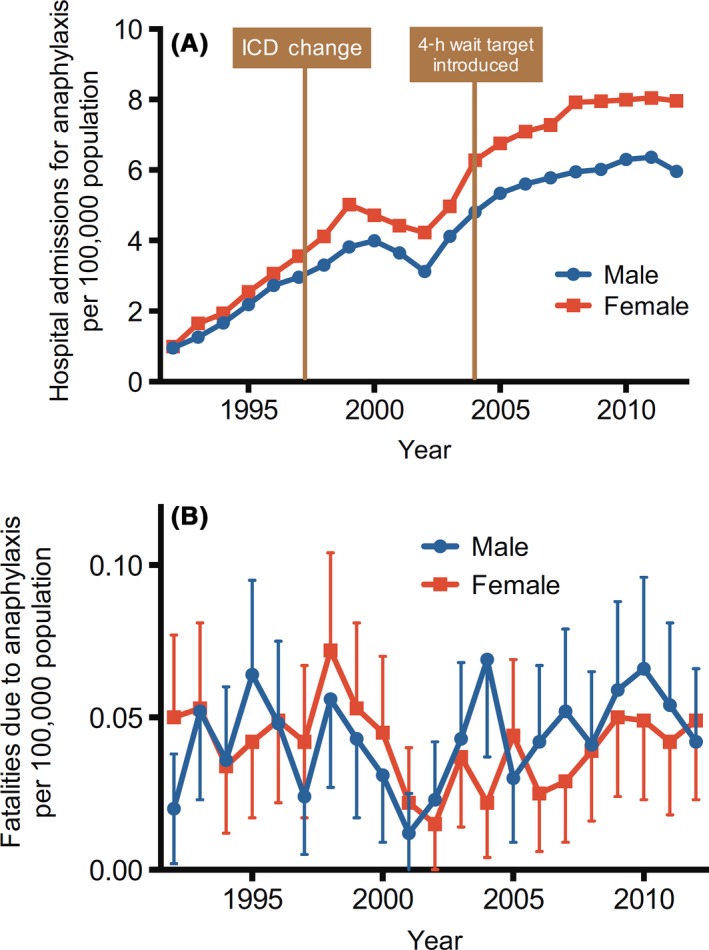
Time trends of hospital admissions (A) and fatalities (B) for all‐cause anaphylaxis in the UK between 1992 and 2012. Vertical bars represent standard error of the means.^6^

**Figure 2:**
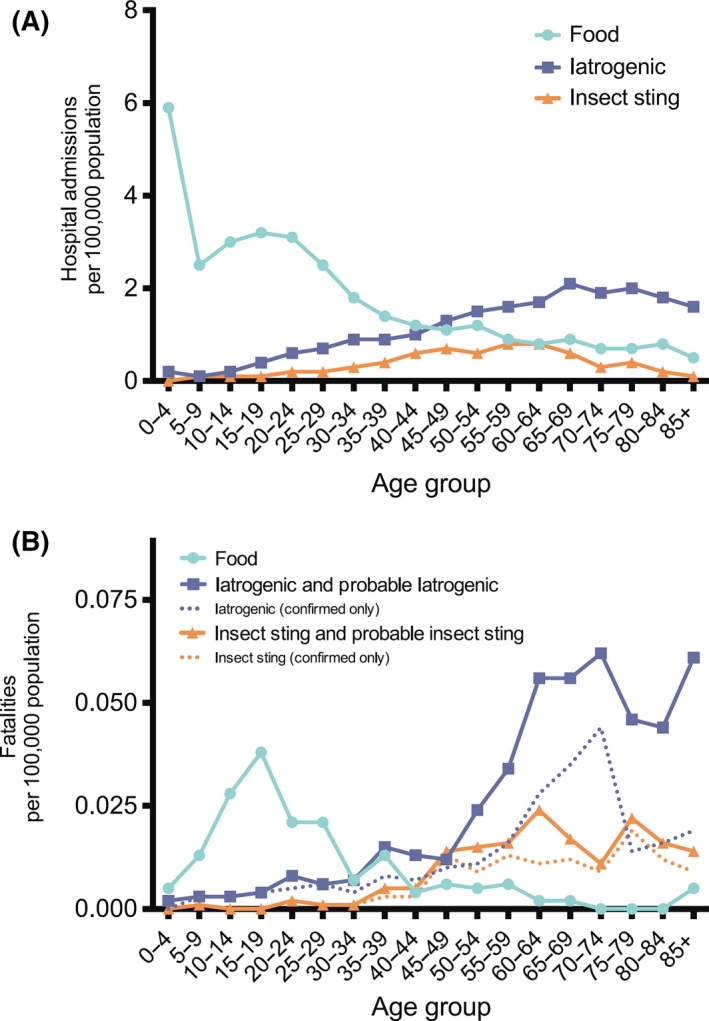
Age‐standardized rates of admission (1998–2012; A) and fatalities (1992‐2012; B) due to food‐related anaphylaxis relative to other causes (iatrogenic causes and insect stings).^6^

The incidence rate of anaphylaxis has increased during the last decade. Reportedly, in Australia, the increase over the last decade may be as high as 350% for food‐induced anaphylaxis and 230% for non‐food‐induced anaphylaxis (Table 1).^5^ Hospital admissions from all‐cause anaphylaxis in England and Wales increased by 615% over the 10‐year study period (1992–2012) (Fig. 1A).^6^ In contrast, fatality rates from all‐cause anaphylaxis have remained stable, at a mean of 0.047 cases per 100,000 population per annum, with no increase in fatalities during this period (Fig. 1B). Although the ratio of patients with anaphylaxis to all patients admitted to the ER was reportedly 0.08%,^4^ this ratio was likely to have been underestimated due to the difficulty in diagnosing anaphylaxis. In general, foods are the most common cause of anaphylaxis, although there is a wide variation in the ratio of food‐/non‐food‐induced anaphylaxis among studies.^4^, ^6^, ^7^, ^8^, ^9^ An important factor affecting the etiology of anaphylaxis is patient age.^6^, ^10^, ^11^, ^12^ For example, foods are the most common cause of anaphylaxis in patients below 44 years of age, whereas iatrogenic causes, mostly drugs, are the most common causes in patients over 45 years of age (Fig. 2A).^6^ This difference may simply reflect the fact that the elderly have a greater opportunity for exposure to medications than young people. Indeed, the risk of drug‐induced anaphylaxis (DIA) increases with age, and is most likely related to the increased use of multiple drugs.^10^ However, the role of other host risk factors for DIA, including that of atopy, remains unclear, and published reports suggest that the majority of patients with fatal DIA had no prior indication of their drug hypersensitivity.^10^, ^13^ Hence, there is as yet no specific host risk factor for DIA other than old age. The relatively high mortality following anaphylaxis due to iatrogenic causes should be noted here (Fig. 2B). This may again be a result of the high age of the patients with DIA. A few studies have detailed the causative drugs of anaphylaxis.^4^ Of these, non‐steroidal anti‐inflammatory drugs are the most common offenders, followed by β‐lactams. In addition, there have been an increasing number of reports of anaphylaxis with biological modifiers, including cetuximab and etanercept.^14^ Details of food‐induced anaphylaxis, including the causative foods, are beyond the scope of this review. Insect stings are the third most common cause of anaphylaxis in all age groups (Fig. 2A).

**Table 1:** Summary of studies regarding the epidemiology of anaphylaxis

Author, location, year of publication	Study period	Age group	No. of patients included	Gender ratio, F : M	Clinical symptoms	No. of patients with anaphylaxis	Causative agent	Ref. no.
Alvarez‐Perea *et al*., Spain, 2015	2009–2010	>15 years	116	57:59	Skin/mucosal (98.3%) Respiratory (79.3%) Gastrointestinal (31%)	Foods 15 (25%)^a^Drugs 25 (41.7%)^a^	NSAIDS (56%)^a^β‐lactams (28%)^a^Other (16%)^a^	4
Turner *et al*., UK, 2015	1998–2012	All ages	25,524	NA	NA	Foods 14,675 (57.5%)Drugs 8,161 (32.0%)Insects 2,688 (10.5%)	NA	6
Huang *et al*., USA, 2012	2004–2008	<18 years	213	104:109	NA	Foods 152 (71%)Drugs 19 (9%)Unknown 32 (15%)	NA	7
Beyer *et al*., Germany, 2012	2008–2010	All ages	295	Adults: women, 61.3%; Children and adolescents, male (73.1%)	Skin/mucosal (81.0%)Respiratory tract (74%)Cardiovascular (86.65%)Gastrointestinal (31.6%)	Foods (32.2%)Drugs (29.2%)Insect venom (19.3%)	NA	8
Brown *et al*., Australia, 2001	1998–1999	≥13 years	142	3:2	NA	Drugs (28%)Insects (17.5%)Foods (17%)	NA	9

a For the number of patients with anaphylaxis and causative agents in reference (Ref.) 4, only 60 patients who had final diagnosis in the allergy department were included.

F, female; M, male; NA, not applicable; NSAID, non‐steroidal anti‐inflammatory drug.

## Mechanisms of Anaphylaxis

**Figure 3:**
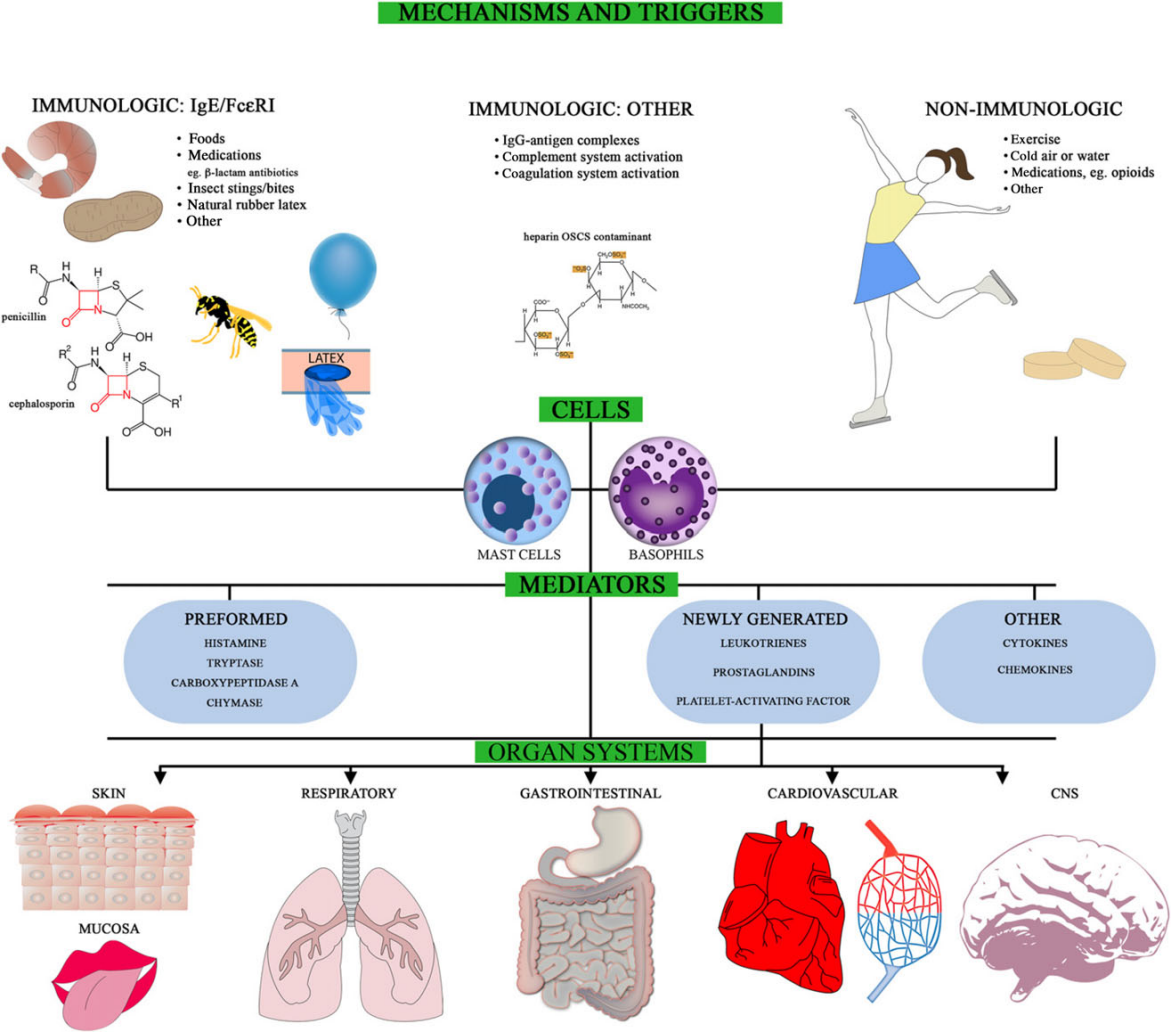
Pathogenesis of anaphylaxis: mechanisms and triggers, cells, mediators, and organ systems affected.^16^ CNS, central nervous system; Ig, immunoglobulin.

A good understanding of the mechanisms of anaphylaxis is essential for making a proper diagnosis (Fig. 3). Anaphylaxis mediated by immunoglobulin (Ig)E, IgG, complement, or immune complexes is defined as immune‐mediated anaphylaxis, as opposed to non‐allergic anaphylaxis (previously known as an anaphylactoid reaction).^15^ Immune‐mediated anaphylaxis is further divided into IgE‐ and non IgE‐mediated types. Non IgE‐mediated anaphylaxis occurs through several mechanisms, such as IgG mediation, activation of complement by IgG immune complex, and direct activation of complement. Although IgE‐mediated activation of mast cells and basophils is a major mode of anaphylaxis, the mechanism underlying DIA actually depends on the causative agent. For example, β‐lactams, muscle relaxants, and certain types of contrast media can bind to IgE as antigens, whereas anaphylaxis mediated by non‐steroidal anti‐inflammatory drugs is mostly caused by non‐allergic mechanisms.^16^ As a consequence of mast cell and basophil activation by any of the above mechanisms, they release more than 100 chemical mediators of anaphylaxis. Initially, preformed mediators, including histamine, tryptase, carboxypeptidase A3, chymase, and proteoglycans are released.^17^, ^18^ These mediators trigger production of arachidonic acid metabolites, including prostaglandins and leukotrienes, and synthesis of platelet‐activating factor. In addition, an array of cytokines and chemokines are synthesized and released.^16^ The plethora of chemical mediators released subsequently affect the target organs and cause various symptoms, as described in the next section.

## Clinical Presentation

In general, the symptoms of anaphylaxis involve a wide variety of organ systems, including the skin, mainly causing urticaria (80–90% of episodes), respiratory tract (70% of episodes), gastrointestinal tract (30–45% of episodes), cardiovascular system (10–45% of episodes), and central nervous system (10–15% of episodes) (Fig. 3).^19^ Spanish emergency physicians reported that the most common clinical manifestation of anaphylaxis is skin and/mucosal symptoms (98.3%), followed by respiratory (79.3%) and gastrointestinal symptoms (31%) (Table 1).^4^ Another study carried out in German ERs, in contrast, reported a high incidence (86.1%) of symptoms of the cardiovascular system, with tachycardia and hypotension being the most often reported. Concomitant symptoms of the skin and mucosal tissue, respiratory symptoms, and gastrointestinal symptoms were documented in 81.4%, 74.6%, and 31.5% of cases, respectively (Table 1).^8^ The author pointed out that the symptoms in children and young adults are a little different from those in the elderly. For example, generalized pruritus and urticaria, vomiting, and diarrhea were more often reported in children and young adults.^8^ Therefore, emergency physicians should be aware that the clinical signs and symptoms of anaphylaxis vary depending on the age group of the patients.

## Diagnosis

As mentioned above, diagnosis of anaphylaxis in an ER setting is not easy. Indeed, studies have shown that a large percentage of patients (57%) who present to the ER with anaphylaxis are misdiagnosed.^2^, ^3^, ^20^, ^21^ The first line of evidence for diagnosing anaphylaxis includes clinical signs. Although no set of diagnostic criteria for anaphylaxis has 100% sensitivity or specificity, the clinical criteria for diagnosing anaphylaxis proposed by the National Institute of Allergy and Infectious Diseases and the Food Allergy and Anaphylaxis Network have been widely used in many countries.^1^ Anaphylaxis is considered likely if any one of three stipulated criteria is satisfied (Table 2). The accuracy of these criteria was retrospectively evaluated in an ER setting, and they were found to have 97% sensitivity and 82% specificity, which in turn means that the negative predictive value was 98% and the positive predictive value was 69%.^3^ These data suggest that a differential diagnosis is necessary even in patients who satisfy these criteria (Table 3). Cutaneous symptoms are key for diagnosing anaphylaxis. Indeed, an international survey including physicians and health professionals as targets indicated that 80% of responders correctly identified cases of anaphylaxis with prominent skin and respiratory symptoms; however, only 55% correctly identified anaphylaxis cases without skin symptoms.^22^ Importantly, the absence of skin manifestations does not preclude a diagnosis of anaphylaxis.^23^, ^24^ Depending on the severity of the reaction, four grades of immediate clinical manifestations are described: grade 1, cutaneous signs only; grade 2, measurable but not life‐threatening symptoms and cutaneous signs, hypotension, tachycardia, and respiratory disturbances, such as cough and difficulty in lung inflation; grade 3, life‐threatening symptoms including collapse, tachycardia or bradycardia, arrhythmias, and bronchospasm; grade 4, cardiac and/or respiratory arrest.^25^

**Table 2:** Clinical criteria for diagnosing anaphylaxis^1^

Anaphylaxis is highly likely when any one of the following three criteria is fulfilled:
1. Acute onset of an illness (within minutes to several hours) with involvement of the skin, mucosal tissue, or both (e.g., generalized hives, pruritus or flushing, swollen lips/tongue/uvula)
And at least one of the following:
a. Respiratory compromise (e.g., dyspnea, wheeze/bronchospasm, stridor, reduced PEF, hypoxemia)
b. Reduced BP or associated symptoms of end‐organ dysfunction (e.g., hypotonia [collapse], syncope, incontinence)
2. Two or more of the following that occur rapidly after exposure to a likely allergen for that patient (minutes to several hours):
a. Involvement of the skin‐mucosal tissue (generalized hives, itch/flush, swollen lips/tongue/uvula)
b. Respiratory compromise (e.g., dyspnea, wheeze/bronchospasm, stridor, reduced PEF, hypoxemia)
c. Reduced BP or associated symptoms (e.g., hypotonia [collapse], syncope, incontinence)
d. Persistent gastrointestinal symptoms (e.g., crampy abdominal pain, vomiting)
3. Reduced BP after exposure to a known allergen for that patient (minutes to several hours):
a. Infants and children: low systolic BP (age specific) or greater than 30% decrease in systolic BP^a^
b. Adults: systolic BP of less than 90 mmHg or greater than 30% decrease from that person's baseline

a Low systolic blood pressure (BP) in children is defined according to age: 1 month–1 year, less than 70 mmHg; 1–10 years, less than [70 mmHg + (2 × age)]; and 11–17 years, less than 90 mmHg.

PEF, peak expiratory flow.

**Table 3:** Differential diagnosis of anaphylaxis^16^

Common entities	Non‐organic disease
Acute generalized hives	Vocal cord dysfunction
Acute asthma	Munchausen syndrome
Syncope (fainting)	
Panic attack	Other forms of shock
Aspiration of a foreign body	Hypovolemic
	Cardiogenic
Restaurant syndromes	Distributive
Monosodium glutamate Sulfites Scombroidosis	Septic (might involve all of the above) Other (e.g., spinal cord injury)
	
	Miscellaneous
Excess endogenous histamine	Non‐allergic angioedema
Mastocytosis/clonal mast cell disorder	Urticarial vasculitis Hyper‐IgE, urticaria syndrome
Basophilic leukemia	
Hydatid cyst	Progesterone anaphylaxis
	Pheochromocytoma
Flush syndromes	Red man syndrome
Perimenopause	Capillary leak syndrome
Carcinoid Autonomic epilepsy Thyroid medullary carcinoma	Cardiovascular (myocardial infarction) Neurologic events (seizure, cerebrovascular event)

IgE, immunoglobulin E.

The second line of evidence for diagnosing anaphylaxis is biological assessment, including plasma histamine and tryptase measurements. The diagnostic accuracy of these assays is increased when histamine and tryptase measurements are combined.^26^ Histamine is a preformed inflammatory mediator contained in the granules of mast cells and basophils, and is released during allergic and non‐allergic reactions. Conversely, absence of an increase in histamine levels does not preclude an immunologic or non‐immunologic anaphylactic reaction.^27^ An early increase in plasma histamine concentration indicates activation of mast cells and/or basophils. Plasma histamine levels are increased for only 15–60 min after symptom onset.^28^ Due to the short plasma half‐life of histamine, measurements should ideally be carried out within 15 min after the reaction for isolated mucocutaneous reactions (grade 1), within 30 min for grade 2 reactions, and within 2 h for more severe reactions.^26^ Tryptase is a mast cell neutral serine protease and a preformed enzyme. Two major forms of tryptase have been identified: α‐tryptase, which is secreted constitutively and is increased in mastocytosis, and β‐tryptase, which is preferentially stored in mast cell granules and, when systemically released, reflects mast cell activation with mediator release. Serum tryptase concentrations reach a peak between 15 min and 1 h after a reaction and decline under first‐order kinetics with a half‐life of approximately 2 h.^28^, ^29^ An increase in total tryptase concentrations (i.e., the sum of α‐ and β‐tryptase) is highly suggestive of mast cell activation, as seen in anaphylaxis, but its absence does not preclude the diagnosis. In fact, elevated histamine and, less commonly, elevated tryptase levels are observed in almost 50% of patients presenting to the ER with acute allergic reactions.^30^ The available evidence suggests that an increased concentration of histamine without elevation of tryptase may be due to an immediate allergic or non‐allergic hypersensitivity reaction activated exclusively by basophils.^26^ Given the relatively short half‐life of histamine and tryptase, collecting blood samples at a proper time is important for reducing false‐negative reactions.

## Initial Treatment of Anaphylaxis

### First‐line intervention

**Figure 4:**
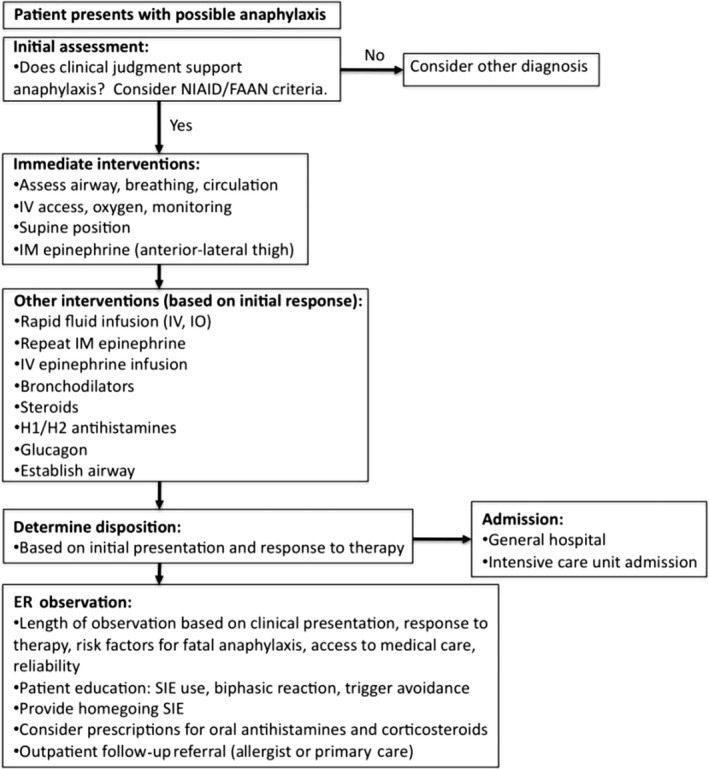
Emergency anaphylaxis management algorithm.^14^ ER, emergency room; FAAN, Food Allergy and Anaphylaxis Network; NIAID, National Institute of Allergy and Infectious Diseases; SIE, self‐injecting epinephrine.

**Figure 5:**
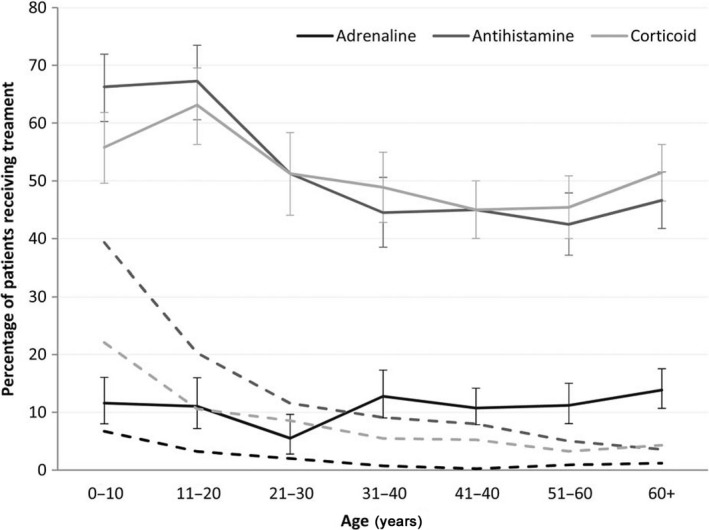
Drugs used for emergency treatment of anaphylaxis, according to age.^37^ The dashed lines indicate the proportion of patients who received only inhalation (adrenaline) or oral (antihistamine or corticoid) treatment. Error bars indicate 95% confidence intervals.

There are several guidelines for the treatment of patients with symptoms of anaphylaxis. Adrenaline is the only drug recommended as first‐line therapy in all published national anaphylaxis guidelines (Fig. 4).^1^, ^31^, ^32^, ^33^ Early injection of adrenaline for anaphylaxis, defined as injection before ER arrival, can significantly reduce the likelihood of hospital admission, as compared with initial injection after arrival at the ER.^34^ Although no human studies regarding the timing of treatment for anaphylaxis could be found, analysis of 92 deaths related to anaphylaxis showed that adrenaline was given prior to cardiac arrest in only 22 of the cases (24%).^35^ This evidence supports the importance of early adrenaline injection. The beneficial effect of adrenaline results from its action on systemically distributed adrenaline receptors.^32^ Its α1 agonist vasoconstrictor effects prevent and relieve airway edema, hypotension, and shock; its β1 agonist chronotropic and ionotropic effects increase the rate and force of cardiac contractions, and its β2 agonist effects lead to bronchodilation. More importantly, β2 adrenergic receptor activation reduces the total amount of mediator released from immune cells. Most international anaphylaxis guidelines recommend injection of adrenaline by the intramuscular route in the mid‐anterolateral thigh at a dose of 0.01 mg/kg of a 1:1,000 (1 mg/mL) solution, up to a maximum of 0.5 mg in adults (0.3 mg in children). Depending on the severity of the episode and the response to the initial injection, the dose can be repeated every 5–15 min, as required.^1^, ^31^, ^33^, ^36^ Intravenous adrenaline is an option in patients with severe hypotension or cardiac arrest unresponsive to intramuscular doses of adrenaline and fluid resuscitation.^1^ Despite these therapeutic benefits of adrenaline and the recommendations for its use, adrenaline injection rates remain low in many ERs^37^, ^38^ (Fig. 5). Researchers have pointed out several possible reasons for the inadequate or lack of use of adrenaline in clinical settings, including: (i) limited knowledge of the treatment algorithm for anaphylaxis, (ii) lack of experience with the use of adrenaline outside the cardiac arrest setting, (iii) reluctance to treat a tachycardiac patient with a drug with positive chronotropic effects.^39^ Physicians should have knowledge about the adverse effects of adrenaline in order to minimize them. The safety profile of intramuscular adrenaline is excellent, although patients may experience transient pallor, palpitations, and headache. However, serious adverse effects, such as ventricular arrhythmias, hypertensive crisis, and pulmonary edema can potentially occur after i.v. administration of an overdose of adrenaline.^31^ A recent study showed that the risks of overdose and adverse cardiovascular events were significantly higher with i.v. bolus administration of adrenaline.^40^ Physician confusion about the correct adrenaline dose and route of administration for the initial treatment of anaphylaxis versus that for shock and cardiac arrest can result in fatal epinephrine overdose.^41^ It should be emphasized that treatment must be tailored according to the clinical severity of the symptoms, the patient's history, and response to emergency treatment.^26^ In addition to adrenaline injection, supplemental oxygen should be administered by face mask or an oropharyngeal airway at a flow rate of 6–8 L/min to all patients with respiratory distress and those receiving repeated doses of adrenaline (Fig. 4). In addition, aggressive fluid resuscitation helps to counteract the significant plasma leak associated with anaphylaxis and complements parenteral adrenaline therapy. In the early stages of anaphylaxis, children might require successive i.v. fluid boluses of 20 mL/kg and adults might require successive i.v. boluses of 1,000 mL to maintain blood pressure.^14^ Finally, patients with anaphylaxis should not suddenly sit, stand, or be placed in the upright position. Instead, they should lie in the supine position with their lower extremities elevated or, if they are experiencing respiratory distress or vomiting, they should be placed in a position of comfort with their lower extremities elevated.^31^

### Second‐line interventions

International guidelines concur that H1 and H2 antihistamines and glucocorticoids are second‐line^31^ and even third line^33^ treatments for anaphylaxis. These medications are not life‐saving and should not be used as initial or sole treatment. Patients chronically medicated with β‐blockers and/or angiotensin‐converting enzyme inhibitors are potentially at risk for development of unresponsiveness to adrenaline.^42^ In such patients, vasopressin,^43^ methylene blue,^44^ and glucagon^33^, ^42^, ^45^ might be effective.

## Biphasic Reactions

Biphasic reactions are characterized by complete clinical resolution of the initial symptoms, followed by the onset of late‐phase symptoms.^46^, ^47^, ^48^ Although the timing of late‐phase symptom onset after resolution of the initial event ranges from 1 to 78 h, most second responses occur within 8 h after resolution of the primary event.^47^ Although there are no consistently reported risk factors, several possible features of the initial episode have been mentioned as predisposing factors for a biphasic reaction. These include: oral administration of antigen, reactions in patients on β‐blockers, reactions in elderly individuals with cardiovascular diseases, delay of more than 30 min between administration of the antigen and appearance of symptoms of the first event, and presence of hypotension or laryngeal edema during the initial event.^47^ Moreover, some reports mentioned treatment differences between patients with uniphasic and biphasic events. These included delay in administration of adrenaline for the primary response, inadequate dose of adrenaline for treatment of the primary reaction, and absence of or too small a dose of corticosteroids given for the initial treatment.^47^, ^49^ Therefore, when anaphylactic shock is identified, the patient should be treated with adrenaline and observed for at least 8 h to rule out a biphasic reaction.

## Management after Discharge

After discharge from the hospital, it is important for patients to be able to cope with possible future anaphylactic episodes (Fig. 4). For this purpose, guidelines for the management of anaphylaxis recommend providing or prescribing adrenaline auto‐injectors to patients at risk of anaphylaxis before discharge from the ER.^14^ In fact, approximately half of the physicians in ERs in the USA reported that >75% of patients seen for anaphylaxis were discharged with a prescription for an adrenaline auto‐injector. However, one‐third of physicians reported that in their ER, ≤50% of patients received such a prescription.^50^ In addition to adrenaline auto‐injectors, follow‐up with a physician with expertise in the diagnosis and management of anaphylaxis, such as an allergist–immunologist, is also important, because they can coordinate additional outpatient testing, provide additional allergen avoidance counselling, develop a detailed emergency action plan for future reactions, and reinforce proper use of the auto‐injectable adrenaline.^14^ Hence, ER physicians should provide or prescribe an adrenaline auto‐injector and act as an intermediary between patients and allergy specialists.

## Confirmation of Anaphylaxis Trigger(s)

Etiological diagnosis, including confirmation of anaphylaxis trigger(s), is essential to avoid future anaphylactic episodes. However, over 40% of patients in ER settings are reportedly discharged without an etiological diagnosis.^4^ Moreover, in 35% of patients with suspected anaphylaxis in the ER, the diagnosis was changed or a suspected trigger was identified after allergy/immunology evaluation.^21^ Given these evidences, emergency physicians should manage allergist follow‐ups after the patient's ER visit to enable an etiological diagnosis, and encourage patients to undergo appropriate tests for confirmation of the anaphylaxis trigger(s). Skin tests, which involve exposure of the mast cells in the skin of patients who experience anaphylaxis to the suspected allergen, remain the gold standard for detection of IgE‐mediated reactions.^27^ The optimal time for testing is generally considered to be 3–4 weeks after an acute anaphylactic episode.^31^ The allergist undertaking the relevant tests must be experienced in the management of drug allergies.^26^ Additionally, during anaphylaxis, proteins such as CD63 and CD203c become newly or increasingly expressed on the surface of basophils. These can be detected by flow cytometry, which forms the basis of experimental drug‐induced basophil activation tests.^51^ In future, basophil activation tests may replace skin tests as the gold standard for making a definitive diagnosis of anaphylaxis, as this assay has the advantage of not being associated with the risk of inducing anaphylaxis during the test.^52^ Another test that can be carried out *in vitro* is measurement of drug‐specific IgE levels. As drug‐specific IgE can be detected only in patients with IgE‐mediated anaphylaxis, it can only be adopted in some patients with anaphylactic reactions.

## Conclusions

The initial treatment strategy, followed by correct diagnosis, in the emergency room is critical for preventing fatal anaphylaxis. Despite the therapeutic benefits of adrenaline and the recommendations for its use, adrenaline injection rates to treat anaphylaxis remain low in many ERs. Emergency room physicians should have proper knowledge regarding the diagnosis and treatment of anaphylaxis.

## Conflict of interest

None declared.
